# Marijuana use is inversely associated with liver steatosis detected by transient elastography in the general United States population in NHANES 2017–2018: A cross-sectional study

**DOI:** 10.1371/journal.pone.0284859

**Published:** 2023-05-18

**Authors:** Rui Du, Xiao-Yan Tang, Cheng Yang, Wen-Hong Gao, Shun-Ji Gao, Hui-Juan Xiang, Li Yang

**Affiliations:** 1 Department of Ultrasound, General Hospital of Central Theater Command, Wuchangqu, Wuhan, Hubei, China; 2 Department of Cardiology, General Hospital of Central Theater Command, Wuchangqu, Wuhan, Hubei, China; 3 Department of Radiology, General Hospital of Central Theater Command, Wuchangqu, Wuhan, Hubei, China; Tower Health - Phoenixville Hospital, UNITED STATES

## Abstract

**Background:**

The impact of marijuana on the general population is largely unknown. The present study aimed to assess the association between marijuana use and liver steatosis and fibrosis in the general United States population utilizing data from the National Health and Nutrition Examination Survey (NHANES).

**Methods:**

This cross-sectional study was performed with data from the 2017–2018 cycle of NHANES. The target population comprised adults in the NHANES database with reliable vibration controlled transient elastography (VCTE) results. The median values of the controlled attenuation parameter (CAP) and liver stiffness measurement (LSM) were used to evaluate liver steatosis and fibrosis, respectively. After adjusting for relevant confounders, a logistic regression analysis was used to assess the association between marijuana use and liver steatosis and fibrosis.

**Results:**

A total of 2622 participants were included in this study. The proportions of never marijuana users, past users, and current users were 45.9%, 35.0%, and 19.1%, respectively. Compared to never marijuana users, past and current users had a lower prevalence of liver steatosis (*P* = 0.184 and *P* = 0.048, respectively). In the alcohol intake-adjusted model, current marijuana use was an independent predictor of a low prevalence of liver steatosis in people with non-heavy alcohol intake. The association between marijuana use and liver fibrosis was not significant in univariate and multivariate regression.

**Conclusion:**

In this nationally representative sample, current marijuana use is inversely associated with steatosis. The pathophysiology is unclear and needs further study. No significant association was established between marijuana use and liver fibrosis, irrespective of past or current use.

## 1. Introduction

Marijuana use has increased dramatically across the United States because several states have legalized its use for recreational and medicinal purposes [1]. The use of marijuana is well documented in the general population, as is its medicinal potential in controlling the symptoms of diseases such as human immunodeficiency virus (HIV), cancer, and bowel diseases [2–5]. Some effects of marijuana on nonalcoholic fatty liver disease (NAFLD) [6] and chronic liver disease [7] have been reported. However, the exact effects of marijuana and its derivates are incompletely elucidated on the general adult population, and little is known about their effects on steatosis and fibrogenesis. Currently, data from animal model studies conflict with whether marijuana contributes to or protects against fibrosis [8].

In this study, we sought to characterize the general adult population with reported marijuana use undergoing vibration controlled transient elastography (VCTE) and evaluate the association between reported marijuana use and liver steatosis and fibrosis by VCTE.

## 2. Materials and methods

### 2.1. Description of data and preprocessing

The analysis data were obtained from the National Health and Nutrition Examination Survey (NHANES) database 2017–2018, conducted in the US by the National Center for Health Statistics of the Centers for Disease Control and Prevention.

NHANES employs a stratified, multistage, clustered probability sampling design to obtain a representative sample of the non-institutionalized civilian population in the US. All participant-related data, including demographic variables, biochemical parameters, and liver ultrasonography, were collected by well-trained examiners. Detailed methods of collecting data are described elsewhere [9]. The Center for Disease Control and Prevention Research Ethics Review Board approved the original investigation and written informed consent was obtained from all participants. The current study was approved by the Medical Ethics Committee of General Hospital of Central Theater Command.

### 2.2. Marijuana use

In NHANES 2017–2018, the drug questionnaire focused on the lifetime and current use of marijuana. The questionnaire was self-completed during the mobile examination center (MEC) interview using the Audio Computer-Assisted Self-Interview system. The following questions defined marijuana use: (1) "Have you ever, even once, used marijuana or hashish?" (2) "How long has it been since you last used marijuana or hashish?" (3) "During the past 30 days, on how many days did you use marijuana or hashish?" Marijuana use was classified as the past user (lifetime use at least once, but not in the past 30 days.) and current user (≥1 day in the last 30 days). The reference group was never user (no lifetime use).

### 2.3. Liver vibration controlled transient elastography

The FibroScan^®^ Model 502 V2 Touch (Echosens, Paris, France) was equipped with a medium (M) and extra-large (XL) wand probe for performing VCTE, a fully validated non-invasive technique used to assess liver steatosis (by controlled attenuation parameter; CAP) and fibrosis (by liver stiffness measurement; LSM) [10–12]. Elastography examinations were performed by NHANES health technicians, trained and certified by NHANES employees and equipment manufacturer (Echosens^TM^, USA). The examinations were carried out following the manufacturer’s guidelines.

The M probe was used initially unless the machine indicated the use of the XL probe. For the present analysis, exams were considered reliable only if at least 10 LSM values were obtained after fasting for at least 3 h, with an interquartile range (IQR)/median <30%. The median CAP values ≥274 (based on a 90% sensitivity cutoff), 290, and 302 dB/m indicated S1, S2, and S3 steatosis, respectively, in accordance with recent studies by Eddowes et al. and Ciardullo et al. [13, 14]. A median LSM ≥8.2 kPa indicated significant fibrosis (F2), whereas values ≥9.7 and 13.6 kPa indicated advanced fibrosis (F3) and cirrhosis (F4), respectively.

### 2.4. Laboratory tests and clinical data

Height (cm) and weight (kg) were ascertained during the MEC visit. Body mass index (BMI) was calculated as weight (kg)/height (m^2^) and classified into the following WHO classes [15]: Normal weight (<25), Overweight (25−30), and Obese (≥30). Hypertension was defined as systolic blood pressure (SBP) value ≥140 mmHg and/or diastolic blood pressure (DBP) ≥90 mmHg or currently taking antihypertensive drugs [16]. Diabetes was defined if any of the following conditions were fulfilled: 1) A self-reported diagnosis of diabetes; 2) Use of anti-diabetic drugs; 3) Hemoglobin A1c (HbA1c) level ≥6.5% (48 mmol/mol); 4) Fasting plasma glucose level ≥126 mg/dL; 5) Random plasma glucose level ≥200 mg/dL [17]. Cardiovascular disease (CVD) was defined as the combination of coronary artery disease and stroke or transient ischemic attacks. Chronic kidney disease (CKD) was defined according to the KDIGO 2021 Clinical Practice Guideline for the Management of Glomerular Diseases [18]. Education level was divided into high school or lower *vs*. college or above. The family income-to-poverty ratio categorized as ≤0.99 is below the poverty level, while ≥1.00 is at or above the poverty level. Smoking status was classified as never smoker, former smoker, and current smoker. Alcohol consumption was calculated using self-reported data on the amount and frequency of alcohol, classified as [19]: 1) never-to-moderate drinker (<2 drinks/day for females, <3 drinks/day for males, or binge drinking <2 days/month); 2) heavy drinker (≥2 drinks/day for females, ≥3 drinks/day for males, or binge drinking ≥2 days/month). Hepatitis C virus infection was indicated by the presence of viral RNA and/or a confirmed antibody test and hepatitis B virus infection as a positive surface antigen test.

### 2.5. Analysis sample

A total of 5569 participants aged ≥20 years attended the MEC visit. Initially, we excluded 304 participants without sufficient data on MEC. Then, 225 individuals were considered ineligible for VCTE for various reasons (unable to lie down, currently pregnant, implanted electronic medical device), and 136 additional individuals were excluded since VCTE was not conducted due to refusal, insufficient time for the examination, and other physical or technical limitations.

**Fig 1:**
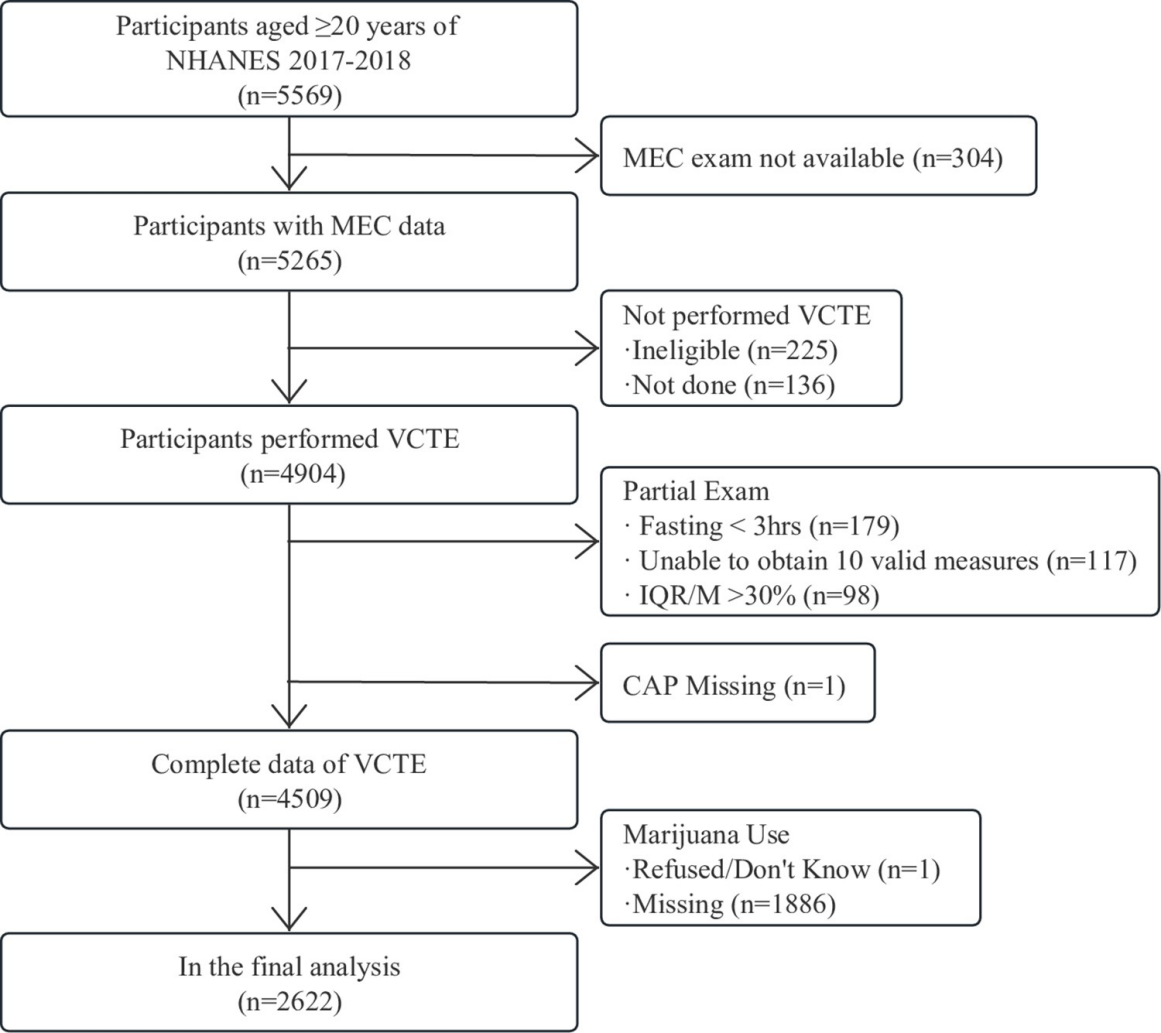
Flowchart of the study.

Among the remaining 4904 participants, 395 (8.1%) had an incomplete VCTE exam because of fasting for <3 h (n = 179), inability of obtaining 10 valid measures (n = 117), an IQR/median LSM value ≥30% (n = 98), and missing CAP (n = 1). Finally, participants who refused marijuana use or had no marijuana use data (n = 1887) were excluded, leading to a final sample of 2622 participants (Fig 1).

### 2.6. Statistical analysis

Measurement data were expressed as mean±standard deviation (x±SD), median (P_25_, P_75_), or N (%). Differences among groups were analyzed using Student’s t-test or one-way analysis of variance (ANOVA), Student-Newman-Kauls (SNK), or the least significant difference (LSD) method was used for multiple comparisons. Qualitative data were described as percentages and analyzed using the chi-square (c^2^) test or Fisher’s exact test as indicated. Multivariate Logistic regression analysis were set up to test the association of marijuana use with steatosis and fibrosis. A biological plausibility approach was followed for the choice of predictors, including known risk factors for steatosis and fibrosis, such as demographic, lifestyle, health status, and physical measurements. The crude model was no covariables adjusted. In model I, covariables were adjusted for age and sex. In model II, covariables were further adjusted for BMI, ethnicity, education level, marital status, poverty level, smoking status, and alcohol intake. In model III, covariables were further adjusted for CVD, CKD, COPD, hypertension, diabetes, anemia, hepatitis B, and hepatitis C. Subgroup analyses were performed to examine the relationship between marijuana use and liver steatosis in different subgroups, including never and mild alcohol consumption group, and moderate and heavy alcohol consumption group. The *P*-value was two-sided, and <0.05 indicated a statistically significant. All the analyses were performed using the statistical software package R (http://www.R-project.org, The R Foundation) and Free statistics software version 1.7.

## 3. Results

### 3.1. Baseline characteristics of study subjects

A total of 2622 participants (1287 males and 1335 females) qualified for this study. The mean age of the cohort was 40.0±11.7 (range: 20–59) years. The proportion of participants reported as never users or past users was 45.9% and 35.0%, respectively, whereas the proportion of current users was 19.1% of American adults.

In Table 1, a total of 1085 (41.38%) participants had various degrees of steatosis. Briefly, participants with steatosis were more likely to be male, older, Mexican American, and had a higher BMI. Participants who were married or living with a partner, and had hypertension, diabetes, or CVD had a higher prevalence of steatosis. The prevalence of steatosis was 50.3% in participants who never used marijuana, 35% in participants who were past users, and 14.7% in current users.

**Table 1:** Baseline characteristics of the participants.

Characteristics	Total (n = 2622)	No Steatosis (n = 1537)	Steatosis (n = 1085)	*P* value	No Fibrosis (n = 2425)	Fibrosis (n = 197)	*P* value
Age, year	40.0 ± 11.7	38.1 ± 11.6	42.6 ± 11.2	<0.001	39.6 ± 11.6	44.7 ± 10.8	<0.001
**Sex, n (%)**				<0.001			0.008
Male	1287 (49.1)	674 (43.9)	613 (56.5)		1172 (48.3)	115 (58.4)	
Female	1335 (50.9)	863 (56.1)	472 (43.5)		1253 (51.7)	82 (41.6)	
**BMI, n (%)**				<0.001			<0.001
Normal weight	733 (28.1)	662 (43.3)	71 (6.6)		713 (29.6)	20 (10.2)	
Over weight	789 (30.3)	498 (32.6)	291 (27)		769 (31.9)	20 (10.2)	
Obese	1085 (41.6)	369 (24.1)	716 (66.4)		929 (38.5)	156 (79.6)	
**Ethnicity, n (%)**				<0.001			0.063
Mexican American	408 (15.6)	171 (11.1)	237 (21.8)		368 (15.2)	40 (20.3)	
Other Hispanic	243 (9.3)	151 (9.8)	92 (8.5)		226 (9.3)	17 (8.6)	
Non-Hispanic White	818 (31.2)	484 (31.5)	334 (30.8)		753 (31.1)	65 (33)	
Non-Hispanic Black	594 (22.7)	393 (25.6)	201 (18.5)		555 (22.9)	39 (19.8)	
Non-Hispanic Asian	397 (15.1)	240 (15.6)	157 (14.5)		378 (15.6)	19 (9.6)	
Other	162 (6.2)	98 (6.4)	64 (5.9)		145 (6)	17 (8.6)	
**Education level, n (%)**				0.141			0.129
High school or lower	413 (15.8)	228 (14.8)	185 (17.1)		374 (15.4)	39 (19.8)	
College or above	2208 (84.2)	1308 (85.2)	900 (82.9)		2050 (84.6)	158 (80.2)	
**Marital status, n (%)**				<0.001			0.113
Married or Living with partner	1559 (59.5)	862 (56.1)	697 (64.2)		1437 (59.3)	122 (61.9)	
Widowed or Divorced	368 (14.0)	204 (13.3)	164 (15.1)		334 (13.8)	34 (17.3)	
Never married	694 (26.5)	470 (30.6)	224 (20.6)		653 (26.9)	41 (20.8)	
**Poverty level, n (%)**				0.428			0.468
Low	441 (19.0)	265 (19.6)	176 (18.2)		403 (18.8)	38 (21.3)	
High	1878 (81.0)	1087 (80.4)	791 (81.8)		1738 (81.2)	140 (78.7)	
**Marijuana use, n (%)**				<0.001			0.221
Never	1204 (45.9)	658 (42.8)	546 (50.3)		1105 (45.6)	99 (50.3)	
Past user	917 (35.0)	537 (34.9)	380 (35)		848 (35)	69 (35)	
Current user	501 (19.1)	342 (22.3)	159 (14.7)		472 (19.5)	29 (14.7)	
**Smoking status, n (%)**				<0.001			0.22
Never smoker	1623 (61.9)	973 (63.3)	650 (59.9)		1512 (62.4)	111 (56.3)	
Former smoker	441 (16.8)	221 (14.4)	220 (20.3)		401 (16.5)	40 (20.3)	
Current smoker	558 (21.3)	343 (22.3)	215 (19.8)		512 (21.1)	46 (23.4)	
**Alcohol intake, n (%)**				0.15			0.864
Never-to-moderate drinker	1141 (50.3)	660 (49)	481 (52.1)		1060 (50.2)	81 (50.9)	
Heavy drinker	1128 (49.7)	686 (51)	442 (47.9)		1050 (49.8)	78 (49.1)	
**Comorbidities**							
Hypertension, n (%)				<0.001			<0.001
No	1933 (73.7)	1247 (81.1)	686 (63.2)		1832 (75.5)	101 (51.3)	
Yes	689 (26.3)	290 (18.9)	399 (36.8)		593 (24.5)	96 (48.7)	
Diabetes, n (%)				<0.001			<0.001
No	2141 (81.7)	1391 (90.5)	750 (69.1)		2026 (83.5)	115 (58.4)	
Yes	481 (18.3)	146 (9.5)	335 (30.9)		399 (16.5)	82 (41.6)	
CVD, n (%)				<0.001			0.054
No	2522 (96.2)	1499 (97.5)	1023 (94.3)		2338 (96.4)	184 (93.4)	
Yes	100 (3.8)	38 (2.5)	62 (5.7)		87 (3.6)	13 (6.6)	
CKD, n (%)				<0.001			<0.001
No	2244 (90.1)	1340 (92.5)	904 (86.7)		2107 (91.4)	137 (73.7)	
Yes	247 (9.9)	108 (7.5)	139 (13.3)		198 (8.6)	49 (26.3)	
COPD, n (%)				0.888			0.774
No	2576 (98.2)	1511 (98.3)	1065 (98.2)		2383 (98.3)	193 (98)	
Yes	46 (1.8)	26 (1.7)	20 (1.8)		42 (1.7)	4 (2)	
Anemia, n (%)				0.293			0.988
No	2346 (92.2)	1361 (91.7)	985 (92.9)		2173 (92.2)	173 (92.5)	
Yes	198 (7.8)	123 (8.3)	75 (7.1)		184 (7.8)	14 (7.5)	
Hepatitis B, n (%)				0.379			0.198
No	2611 (99.6)	1532 (99.7)	1079 (99.4)		2416 (99.6)	195 (99)	
Yes	11 (0.4)	5 (0.3)	6 (0.6)		9 (0.4)	2 (1)	
Hepatitis C, n (%)				0.206			0.003
No	2602 (99.2)	1522 (99)	1080 (99.5)		2411 (99.4)	191 (97)	
Yes	20 (0.8)	15 (1)	5 (0.5)		14 (0.6)	6 (3)	

Notes: Data presented are mean±SD or n (%).

Abbreviations: BMI, body mass index

CVD, Cardiovascular disease

CKD, Chronic kidney disease

COPD, chronic obstructive pulmonary disease.

Table 1 also describes the characteristics of samples stratified by liver fibrosis. A total of 197 (7.51%) participants had various degrees of fibrosis, and participants with significant fibrosis were male, older, and had a higher BMI, had higher prevalence of hypertension, diabetes, and hepatitis C.

### 3.2. VCTE results

**Fig 2:**
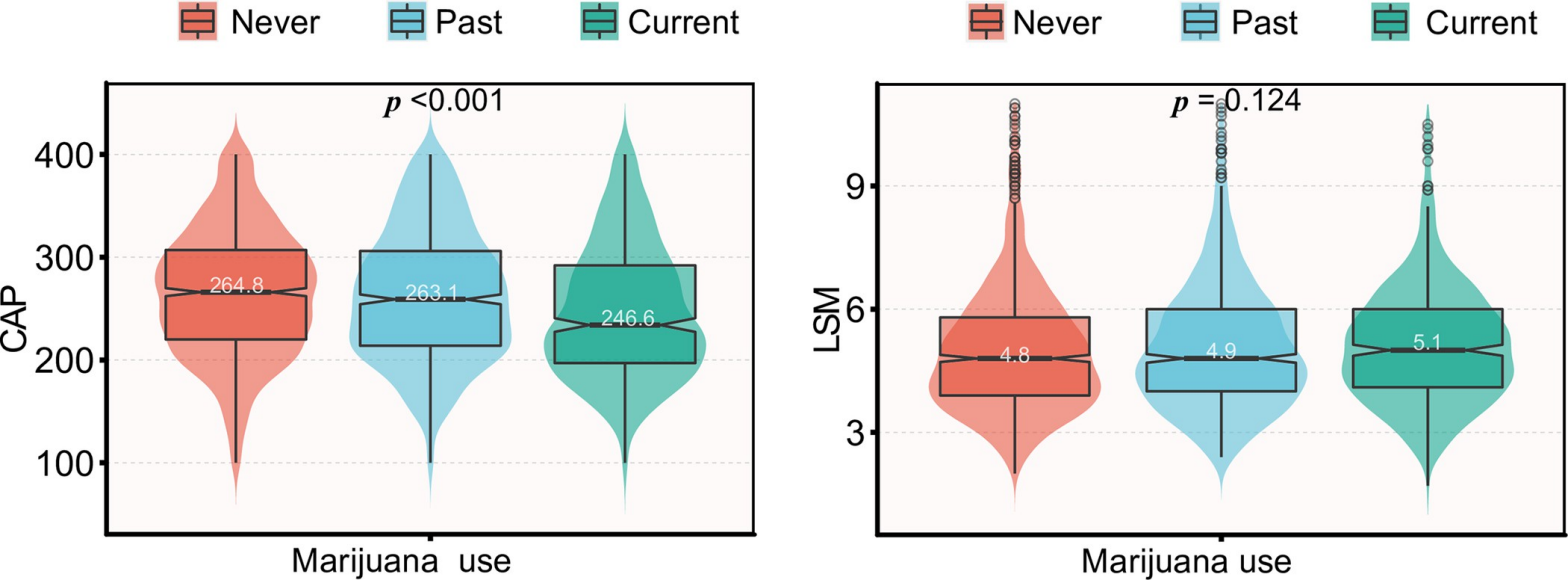
The CAP and LSM of different marijuana use groups. Abbreviations: CAP: controlled attenuation parameter; LSM: liver stiffness measurement.

The VCTE results of the whole participants are shown in Table 2. A total of 1949 (74.3%) participants were evaluated with the M probe and 673 (25.7%) with the XL probe. Marijuana use was associated with a decreasing CAP. In the current user category of marijuana, the CAP was 246.6±64.0 dB/m, while in the past and never user categories, the CAP was 263.1±63.9 dB/m and 264.8±62.2 dB/m, respectively (Fig 2). Various degrees of steatosis were detected in 1085 (41.4%) individuals, and 696 (26.5%) had S3 steatosis, among whom 341 (28.3%) were never users, 256 (27.9%) were past users, 99 (19.8%) were current users.

**Table 2:** Results of VCTE of participants.

Characteristics	Total (n = 2622)	Never (n = 1204)	Past user (n = 917)	Current user (n = 501)	*p*
**Wand type, n (%)**					0.008
M	1949 (74.3)	910 (75.6)	650 (70.9)	389 (77.6)	
XL	673 (25.7)	294 (24.4)	267 (29.1)	112 (22.4)	
CAP (dB/m)	260.8±63.5	264.8±62.2	263.1±63.9	246.6 ± 64.0	<0.001
**Steatosis, n (%)**					<0.001
S0	1537 (58.6)	658 (54.7)	537 (58.6)	342 (68.3)	
S1	227 (8.7)	122 (10.1)	76 (8.3)	29 (5.8)	
S2	162 (6.2)	83 (6.9)	48 (5.2)	31 (6.2)	
S3	696 (26.5)	341 (28.3)	256 (27.9)	99 (19.8)	
LSM (kPa)	4.9 (4.0, 6.0)	4.8 (3.9, 6.0)	4.9 (4.0, 6.1)	5.1 (4.1, 6.0)	0.124
**Fibrosis, n (%)**					0.221
F0-F1	2425 (92.5)	1105 (91.8)	848 (92.5)	472 (94.2)	
≥F2	197 (7.5)	99 (8.2)	69 (7.5)	29 (5.8)	
**Fibrosis, n (%)**					0.499
F0-F1	2425 (92.5)	1105 (91.8)	848 (92.5)	472 (94.2)	
F2	77 (2.9)	37 (3.1)	27 (2.9)	13 (2.6)	
F3	69 (2.6)	32 (2.7)	27 (2.9)	10 (2)	
F4	51 (1.9)	30 (2.5)	15 (1.6)	6 (1.2)	
**LSM quarter, n (%)**					0.211
Q 1	631 (24.1)	310 (25.7)	219 (23.9)	102 (20.4)	
Q 2	654 (24.9)	297 (24.7)	231 (25.2)	126 (25.1)	
Q 3	640 (24.4)	291 (24.2)	211 (23)	138 (27.5)	
Q 4	697 (26.6)	306 (25.4)	256 (27.9)	135 (26.9)	

Notes: Data presented are mean±SD, median (P25, P75) or n (%).

Abbreviations: VCTE, vibration controlled transient elastography.

The LSM of the current user category was 5.1 kPa, while in the past and never user categories, the LSM was 4.9 and 4.8 kPa, respectively. 2425 (92.5%) participants did not have fibrosis, the prevalence of liver fibrosis, advanced fibrosis, and cirrhosis was 77 (2.9%), 69 (2.6%), and 51 (1.9%), respectively; no significant differences were detected between the groups (*P* = 0.499). Stratification by the LSM quartile did not show statistically significant differences between groups (*P* = 0.211).

### 3.3. Marijuana use with steatosis and fibrosis

As shown in Table 3, univariate analysis revealed that age, sex, ethnicity, BMI, marital status, smoking status, marijuana use, hypertension, CVD, CKD, and diabetes were associated with steatosis in participants (all *P*<0.05). Age, sex, ethnicity, BMI, hypertension, CVD, CKD, diabetes, and hepatitis C were associated with fibrosis in participants (all *P*<0.05). However, the association between marijuana use and fibrosis was not significant.

**Table 3:** Unadjusted logistic regression associations of marijuana use and steatosis/fibrosis.

Characteristics	Steatosis		Fibrosis	
	OR (95%CI)	*P*	OR (95%CI)	*P*
**Age**	1.03 (1.03~1.04)	<0.001	1.04 (1.03~1.05)	<0.001
**Sex**				
Male	1		1	
Female	0.6 (0.51~0.7)	<0.001	0.67 (0.5~0.9)	0.007
**Ethnicity**				
Mexican American	1		1	
Other Hispanic	0.44 (0.32~0.61)	<0.001	0.69 (0.38~1.25)	0.222
Non-Hispanic White	0.5 (0.39~0.63)	<0.001	0.79 (0.53~1.2)	0.274
Non-Hispanic Black	0.37 (0.28~0.48)	<0.001	0.65 (0.41~1.02)	0.063
Non-Hispanic Asian	0.47 (0.36~0.63)	<0.001	0.46 (0.26~0.81)	0.007
Other	0.47 (0.33~0.68)	<0.001	1.08 (0.59~1.96)	0.804
**BMI**				
Normal weight	1		1	
Over weight	5.45 (4.1~7.24)	<0.001	0.93 (0.49~1.74)	0.813
Obese	18.09 (13.74~23.82)	<0.001	5.99 (3.72~9.63)	<0.001
**Education level**				
High school or lower	1		1	
College or above	0.85 (0.69~1.05)	0.127	0.74 (0.51~1.07)	0.107
**Marital status**				
Married or Living with partner	1		1	
Widowed or Divorced	0.99 (0.79~1.25)	0.96	1.2 (0.81~1.79)	0.372
Never married	0.59 (0.49~0.71)	<0.001	0.74 (0.51~1.07)	0.106
**Poverty level**				
Low	1		1	
High	1.1 (0.89~1.35)	0.397	0.85 (0.59~1.24)	0.41
**Smoking status**				
Never smoker	1		1	
Former smoker	1.49 (1.21~1.84)	<0.001	1.36 (0.93~1.98)	0.112
Current smoker	0.94 (0.77~1.14)	0.527	1.22 (0.86~1.75)	0.269
**Alcohol intake**				
Never-to-moderate drinker	1		1	
Heavy drinker	0.88 (0.75~1.05)	0.15	0.97 (0.7~1.34)	0.864
**Marijuana use**				
Never	1		1	
Past user	0.85 (0.72~1.01)	0.072	0.91 (0.66~1.25)	0.555
Current user	0.56 (0.45~0.7)	<0.001	0.69 (0.45~1.05)	0.084
**Comorbidities**				
Hypertension				
No	1		1	
Yes	2.5 (2.09~2.99)	<0.001	2.94 (2.19~3.94)	<0.001
CVD				
No	1		1	
Yes	2.39 (1.58~3.61)	<0.001	1.9 (1.04~3.47)	0.037
CKD				
No	1		1	
Yes	1.91 (1.46~2.49)	<0.001	3.81 (2.66~5.44)	<0.001
Diabetes				
No	1		1	
Yes	4.26 (3.44~5.27)	<0.001	3.62 (2.67~4.9)	<0.001
COPD				
No	1		1	
Yes	1.09 (0.61~1.97)	0.771	1.18 (0.42~3.31)	0.759
Anemia				
No	1		1	
Yes	0.84 (0.63~1.14)	0.261	0.96 (0.54~1.68)	0.875
Hepatitis B				
No	1		1	
Yes	1.7 (0.52~5.6)	0.38	2.75 (0.59~12.83)	0.197
Hepatitis C				
No	1		1	
Yes	0.47 (0.17~1.3)	0.145	5.41 (2.06~14.24)	0.001

Notes: OR, odds ratio; 95% CI, 95% confidence interval.

Abbreviations: BMI, body mass index; CVD, Cardiovascular disease; CKD, Chronic kidney disease; COPD, chronic obstructive pulmonary disease.

Table 4 displays a statistically significant association between current marijuana use and steatosis in all multivariate logistic regression models after adjusting for several covariates, including age, sex, BMI, ethnicity, education level, marital status, poverty level, smoking status, alcohol intake, CVD, CKD, COPD, hypertension, diabetes, anemia, hepatitis B, and hepatitis C (Model I: OR = 0.62, 95% CI: 0.49–0.78, *P*<0.001; Model II: OR = 0.7, 95% CI: 0.5–0.98, *P* = 0.039; Model III: OR = 0.69, 95% CI: 0.48–0.997, *P* = 0.048). Furthermore, after adjusting for potential confounding factors according to univariate analysis, the multivariate analysis did not find any significant association between marijuana use and fibrosis.

**Table 4:** Multivariate-adjusted logistic regression associations of marijuana use and steatosis/fibrosis.

	n	Crude		Model I		Model II		Model III	
		OR (95% CI) *P*		OR (95% CI) *P*		OR (95% CI) *P*		OR (95% CI) *P*	
**Steatosis**									
Never	1204	1(Ref)		1(Ref)		1(Ref)		1(Ref)	
Past user	917	0.85 (0.72~1.01)	0.072	0.87 (0.73~1.04)	0.131	0.81 (0.63~1.06)	0.127	0.83 (0.63~1.09)	0.184
Current user	501	0.56 (0.45~0.70)	<0.001	0.62 (0.49~0.78)	<0.001	0.7 (0.5~0.98)	0.039	0.69 (0.48~0.997)	0.048
**Fibrosis**									
Never	1204	1(Ref)		1(Ref)		1(Ref)		1(Ref)	
Past user	917	0.91 (0.66~1.25)	0.555	0.94 (0.68~1.3)	0.712	0.86 (0.56~1.33)	0.503	0.9 (0.57~1.41)	0.64
Current user	501	0.69 (0.45~1.05)	0.084	0.81 (0.52~1.25)	0.338	0.82 (0.46~1.47)	0.509	0.69 (0.36~1.3)	0.248

Model I: Adjusted for age and sex.

Model II: Adjusted for Model I + BMI + ethnicity + education level + marital status + poverty level+ Smoking status + alcohol intake.

Model III: Adjusted for Model II + CVD + CKD + COPD + hypertension + diabetes + anemia+ hepatitis B + hepatitis C.

Notes: OR, odds ratio; 95% CI, 95% confidence interval.

Abbreviations: BMI, body mass index; CVD, Cardiovascular disease; CKD, Chronic kidney disease; COPD, chronic obstructive pulmonary disease.

**Fig 3:**
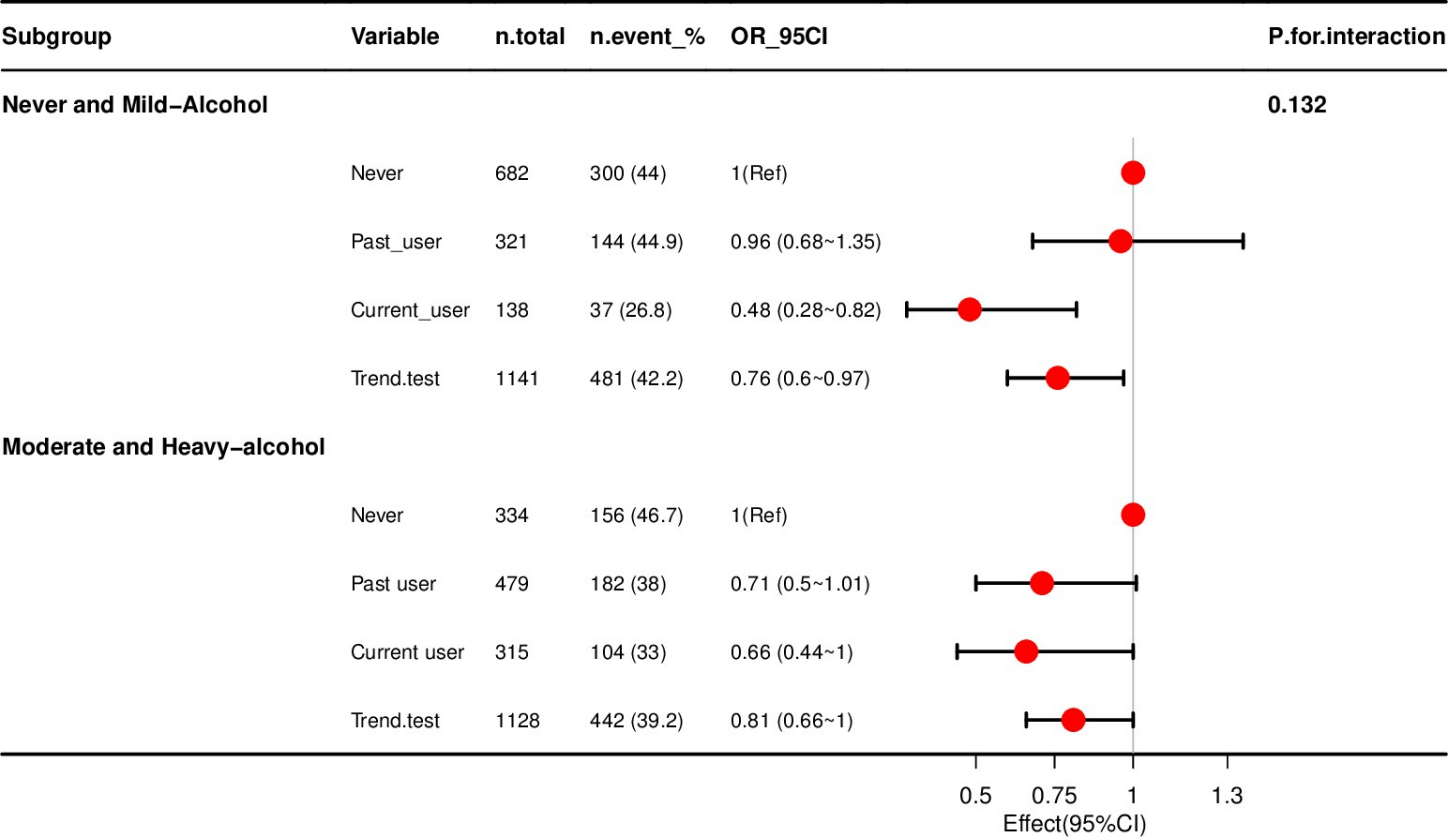
Marijuana use and steatosis according to alcohol intake. Notes: Adjusted for age + sex + BMI + ethnicity + education level + marital status + poverty level + Smoking status + CVD + CKD + COPD + hypertension + diabetes + anemia+ hepatitis B + hepatitis C. OR, odds ratio; 95% CI, 95% confidence interval. Abbreviations: CVD, Cardiovascular disease; CKD, Chronic kidney disease; COPD, chronic obstructive pulmonary disease.

Stratification analysis was carried out in participants. After stratifying the population according to alcohol intake and adjusting for model III, an inverse association was mainly reflected in the subgroups between the current marijuana use and steatosis in participants with non-heavy alcohol intake (Table 5, Fig 3).

**Table 5:** Alcohol intake and steatosis according to marijuana use.

	n	n (%)	Crude OR (95% CI)	*P*	Adj. OR (95% CI)	*P*	*P for interaction*
							0.233
**Never-to-moderate drinker**							
Never	682	300 (44)	1(Ref)		1(Ref)		
Past user	321	144 (44.9)	1.04 (0.79~1.35)	0.795	0.82 (0.55~1.24)	0.348	
Current user	138	37 (26.8)	0.47 (0.31~0.7)	<0.001	0.45 (0.23~0.86)	0.015	
**Heavy drinker**							
Never	334	156 (46.7)	1(Ref)		1(Ref)		
Past user	479	182 (38)	0.7 (0.53~0.93)	0.013	0.83 (0.56~1.24)	0.368	
Current user	315	104 (33)	0.56 (0.41~0.77)	<0.001	0.83 (0.52~1.34)	0.449	

Adjusted for age + sex +BMI + ethnicity + education level + marital status + poverty level + Smoking status + alcohol intake + CVD + CKD + COPD + hypertension + diabetes + anemia+ hepatitis B + hepatitis C.

Abbreviations: BMI, body ***mass*** index; CVD, Cardiovascular disease; CKD, Chronic kidney disease; COPD, chronic obstructive pulmonary disease.

## 4. Discussion

In this national retrospective analysis of the general US adult population with marijuana use and the results of VCTE, the current marijuana use within the past 30 days was associated with decreased steatosis, while past marijuana use had no significant association between marijuana use status and liver steatosis, after accounting for potential confounding variables. According to the current findings, no association was established between marijuana use and liver fibrosis.

Several studies reported that marijuana use is associated with obesity [20], diabetes [21], and gastrointestinal diseases [22]. Hitherto, there has been no consensus on whether marijuana use reduces the prevalence of NAFLD [23, 24]. Kim et al. conducted an observational study and found that active marijuana use protected against NAFLD through serum alanine aminotransferase levels (n = 14080, OR: 0.76; 95% CI: 0.58–0.98, *P* = 0.001 for current light users; OR: 0.70; 95% CI: 0.56–0.89, *P* = 0.001 for current heavy users) and ultrasound diagnosis (n = 8286, OR: 0.77, 95% CI: 0.59–1.00, *P* = 0.053 for current users; OR: 0.71, 95% CI: 0.51–0.97, *P* = 0.033 for current light users [25]. However, these studies lacked data on significant alcohol consumption. The present study also evaluated people who drank alcohol, both past and current drinkers, including heavy drinkers. We extended our findings by showing that marijuana use was still inversely associated with liver steatosis, as described by Adejumo et al. [26].

Marijuana has been shown to have a different effect on the fibrotic progression of some chronic liver diseases. In an observational study of hepatitis C patients in the USA (n = 9456), Adejumo et al. demonstrated that marijuana use was associated with a low prevalence of liver cirrhosis than non-marijuana users and non-dependent users [hazard ratio (HR) 0.81; 95% CI: 0.72–0.91, *P* = 0.0004; HR 0.62; 95% CI: 0.41–0.93, *P* = 0.0156, respectively] [27]. A recent meta-analysis showed that marijuana use did not increase the prevalence or progression of liver fibrosis in HCV and HCV-HIV-coinfected patients (adjusted OR: 0.91, 95% CI: 0.72–1.15, *I*^2^ = 75%) [28]. Herein, we also confirmed that marijuana use was not related to liver fibrosis.

The endocannabinoid system (ECS) has been established as a critical homeostatic regulator that acts through CB-1 and CB-2 receptors, promoting anti-fibrotic and anti-liver fat formation effects, respectively [6, 29]. Marijuana contains >100 active cannabinoids [30] that interact with the same receptors for ECS to produce similar biological effects [31]. The product of marijuana tetrahydrocannabinol decreases the CB1 density and lowers the overall CB1 activity [32]. The other components of marijuana, such as cannabidiol and tetrahydrocannabivarin, reduce the activation of CB1, thereby decreasing liver steatosis and fibrosis [32, 33]. Since fibrosis has a low prevalence in our population, marijuana use might not tip the balance of ECS. Longitudinal follow-up may be required to truly reflect the association of marijuana with fibrosis.

Nevertheless, the present study has several limitations. First, this was an observational study; no causal inference can be made, and correlations should be interpreted as associations. Second, marijuana use was based on self-reporting, and the skewness of the distribution of the number of marijuana use may be subject to misclassification, limiting the power of our secondary analysis with the days of cannabis usage. Such inaccurate reports may introduce a bias towards the null hypothesis for the result. Third, physical activity and diet were not included in the analyses. Furthermore, due to the limitation of the NHANES database, we could not rule out biliary cirrhosis and primary liver diseases such as Wilson’s disease and the use of steatogenic medication. Also, we could not evaluate the type of marijuana and the dose-response correlation between marijuana use *vs*. the prevalence of liver steatosis and fibrosis.

## 5. Conclusions

In conclusion, we found that current marijuana use is inversely associated with liver steatosis. Further studies are required to confirm these results longitudinally, and investigations into marijuana compounds and their biological effects are promising for treating and preventing fatty liver disease.

## Supporting information

S1 Checklist STROBE statement—checklist of items that should be included in reports of observational studies. (DOCX)

S1 Data (CSV)

## Abbreviations

NHANES: National Health and Nutrition Examination Survey
VCTE: vibration controlled transient elastography
CAP: controlled attenuation parameter
LSM: liver stiffness measurement
BMI: body mass index
CVD: Cardiovascular disease
CKD: Chronic kidney disease
NAFLD: nonalcoholic fatty liver disease
ECS: endocannabinoids system
