# Supplementary material for: Marijuana use is inversely associated with liver steatosis detected by transient elastography in the general United States population in NHANES 2017–2018: A cross-sectional study
Source: PLoS One. 2023 May 18;18(5):e0284859. doi: 10.1371/journal.pone.0284859 (PMC10194898; doi:10.1371/journal.pone.0284859)
Supplement: S1 Checklist — (DOCX) [file pone.0284859.s001.docx]

STROBE Statement—checklist of items that should be included in reports of observational studies

|  | Item No. | Recommendation | Page  No. | Relevant text from manuscript |
| --- | --- | --- | --- | --- |
| **Title and abstract** | 1 | (*a*) Indicate the study’s design with a commonly used term in the title or the abstract | 1 | A Cross-sectional Study |
|  |  | (*b*) Provide in the abstract an informative and balanced summary of what was done and what was found | 1,2 | In this nationally representative sample, current marijuana use is inversely associated with steatosis. |
| Introduction | | | |  |
| Background/rationale | 2 | Explain the scientific background and rationale for the investigation being reported | 2 | the exact effects of marijuana and its derivates are incompletely elucidated on the general adult population, and little is known about their effects on steatosis and fibrogenesis. |
| Objectives | 3 | State specific objectives, including any prespecified hypotheses | 2 | we sought to characterize the general adult population with reported marijuana use undergoing vibration controlled transient elastography (VCTE) and evaluate the association between reported marijuana use and liver steatosis and fibrosis by VCTE. |
| Methods | | | |  |
| Study design | 4 | Present key elements of study design early in the paper |  |  |
| Setting | 5 | Describe the setting, locations, and relevant dates, including periods of recruitment, exposure, follow-up, and data collection | 2 | The analysis data were obtained from the National Health and Nutrition Examination Survey (NHANES) database 2017-2018 |
| Participants | 6 | (*a*) *Cohort study*—Give the eligibility criteria, and the sources and methods of selection of participants. Describe methods of follow-up  *Case-control study*—Give the eligibility criteria, and the sources and methods of case ascertainment and control selection. Give the rationale for the choice of cases and controls  *Cross-sectional study*—Give the eligibility criteria, and the sources and methods of selection of participants | 2-4 | The analysis data were obtained from the National Health and Nutrition Examination Survey (NHANES) database 2017-2018, conducted in the US by the National Center for Health Statistics of the Centers for Disease Control and Prevention.  The following questions defined marijuana use. |
|  |  | (*b*) *Cohort study*—For matched studies, give matching criteria and number of exposed and unexposed  *Case-control study*—For matched studies, give matching criteria and the number of controls per case |  |  |
| Variables | 7 | Clearly define all outcomes, exposures, predictors, potential confounders, and effect modifiers. Give diagnostic criteria, if applicable | 3,4 | The median CAP values ≥274, 290, and 302 dB/m indicated S1, S2, and S3 steatosis, respectively. A median LSM ≥8.2 kPa indicated signiﬁcant ﬁbrosis (F2), whereas values ≥9.7 and 13.6 kPa indicated advanced ﬁbrosis (F3) and cirrhosis (F4), respectively. |
| Data sources/ measurement | 8* | For each variable of interest, give sources of data and details of methods of assessment (measurement). Describe comparability of assessment methods if there is more than one group | *3* | The following questions defined marijuana use: (1) "Have you ever, even once, used marijuana or hashish?" (2) "How long has it been since you last used marijuana or hashish?" (3) "During the past 30 days, on how many days did you use marijuana or hashish?" Marijuana use was classified as the past user (lifetime use at least once, but not in the past 30 days.) and current user (≥1 day in the last 30 days). The reference group was never user (no lifetime use). |
| Bias | 9 | Describe any efforts to address potential sources of bias | 8 | marijuana use was based on self-reporting, and the skewness of the distribution of the number of marijuana use may be subject to misclassification, limiting the power of our secondary analysis with the days of cannabis usage |
| Study size | 10 | Explain how the study size was arrived at |  | Fig 1 |

Continued on next page

| Quantitative variables | 11 | Explain how quantitative variables were handled in the analyses. If applicable, describe which groupings were chosen and why | 3,4 | The median CAP values ≥274, 290, and 302 dB/m indicated S1, S2, and S3 steatosis, respectively. A median LSM ≥8.2 kPa indicated signiﬁcant ﬁbrosis (F2), whereas values ≥9.7 and 13.6 kPa indicated advanced ﬁbrosis (F3) and cirrhosis (F4), respectively. |
| --- | --- | --- | --- | --- |
| Statistical methods | 12 | (*a*) Describe all statistical methods, including those used to control for confounding | 4 | Differences among groups were analyzed using Student's t-test or one-way analysis of variance (ANOVA), Student-Newman-Kauls (SNK), or the least significant difference (LSD) method was used for multiple comparisons. |
|  |  | (*b*) Describe any methods used to examine subgroups and interactions | 4,5 | Subgroup analyses were performed to examine the relationship between marijuana use and liver steatosis and ﬁbrosis in different subgroups, including never and mild alcohol consumption group, and moderate and heavy alcohol consumption group. |
|  |  | (*c*) Explain how missing data were addressed |  |  |
|  |  | (*d*) *Cohort study*—If applicable, explain how loss to follow-up was addressed  *Case-control study*—If applicable, explain how matching of cases and controls was addressed  *Cross-sectional study*—If applicable, describe analytical methods taking account of sampling strategy |  |  |
|  |  | (*e*) Describe any sensitivity analyses |  |  |
| Results | | | | |
| Participants | 13* | (a) Report numbers of individuals at each stage of study—eg numbers potentially eligible, examined for eligibility, confirmed eligible, included in the study, completing follow-up, and analysed | 4 | Among the remaining 4904 participants, 395 (8.1%) had an incomplete VCTE exam because of fasting for <3 h (n=179), inability of obtaining 10 valid measures (n=117), an IQR/median LSM value ≥30% (n=98), and missing CAP (n=1). Finally, participants who refused marijuana use or had no marijuana use data (n=1887) were excluded, leading to a final sample of 2622 participants (Fig. 1). |
|  |  | (b) Give reasons for non-participation at each stage | 4 | Among the remaining 4904 participants, 395 (8.1%) had an incomplete VCTE exam because of fasting for <3 h (n=179), inability of obtaining 10 valid measures (n=117), an IQR/median LSM value ≥30% (n=98), and missing CAP (n=1). Finally, participants who refused marijuana use or had no marijuana use data (n=1887) were excluded. |
|  |  | (c) Consider use of a flow diagram |  | Fig 1 |
| Descriptive data | 14* | (a) Give characteristics of study participants (eg demographic, clinical, social) and information on exposures and potential confounders | 5 | A total of 2622 participants (1287 males and 1335 females) qualiﬁed for this study. The mean age of the cohort was 40.0±11.7 (range: 20–59) years. The mean BMI was 29.7±7.5 kg/m^2^. In table 1, a total of 1085 (41.38%) participants had various degrees of steatosis. |
|  |  | (b) Indicate number of participants with missing data for each variable of interest |  |  |
|  |  | (c) *Cohort study*—Summarise follow-up time (eg, average and total amount) |  |  |
| Outcome data | 15* | *Cohort study*—Report numbers of outcome events or summary measures over time |  |  |
|  |  | *Case-control study—*Report numbers in each exposure category, or summary measures of exposure |  |  |
|  |  | *Cross-sectional study—*Report numbers of outcome events or summary measures | *5,6* | Various degrees of steatosis were detected in 1085 (41.4%) individuals, and 696 (26.5%) had S3 steatosis, among whom 341 (28.3%) were never users, 256 (27.9%) were past users, 99 (19.8%) were current users. |
| Main results | 16 | (*a*) Give unadjusted estimates and, if applicable, confounder-adjusted estimates and their precision (eg, 95% confidence interval). Make clear which confounders were adjusted for and why they were included | 6,14 | Table 4 displays a statistically significant association between current marijuana use and steatosis in all multivariate logistic regression models after adjusting for several covariates, including age, sex, ethnicity, education level, marital status, poverty level, smoking status, alcohol intake, CVD, COPD, hypertension, diabetes, anemia, hepatitis B, and hepatitis C (Model I: OR=0.62, 95% CI: 0.49–0.78, P<0.001; Model II: OR=0.6, 95% CI: 0.45–0.81, P=0.001; Model III: OR=0.63, 95% CI: 0.46–0.87, P=0.005) |
|  |  | (*b*) Report category boundaries when continuous variables were categorized | 5 | In the current user category of marijuana, the CAP was 246.6±64.0 dB/m, while in the past and never user categories, the CAP was 263.1±63.9 dB/m and 264.8±62.2 dB/m, respectively (Fig. 2) |
|  |  | (*c*) If relevant, consider translating estimates of relative risk into absolute risk for a meaningful time period |  |  |

Continued on next page

| Other analyses | 17 | Report other analyses done—eg analyses of subgroups and interactions, and sensitivity analyses | 6 | Stratiﬁcation analysis was carried out in participants. After stratifying the population according to alcohol intake and adjusting for model III, an inverse association was mainly reﬂected in the subgroups between the current marijuana use and steatosis in participants (Table 5, Fig. 3). |
| --- | --- | --- | --- | --- |
| Discussion | | | | |
| Key results | 18 | Summarise key results with reference to study objectives | 7 | the current marijuana use within the past 30 days was associated with decreased steatosis, while past marijuana use had no significant association between marijuana use status and liver steatosis, after accounting for potential confounding variables. According to the current findings, no association was established between marijuana use and liver fibrosis. |
| Limitations | 19 | Discuss limitations of the study, taking into account sources of potential bias or imprecision. Discuss both direction and magnitude of any potential bias | 8 | Nevertheless, the present study has several limitations. First, this was an observational study; no causal inference can be made, and correlations should be interpreted as associations. Second, marijuana use was based on self-reporting, and the skewness of the distribution of the number of marijuana use may be subject to misclassification, limiting the power of our secondary analysis with the days of cannabis usage. Such inaccurate reports may introduce a bias towards the null hypothesis for the result. Third, physical activity and diet were not included in the analyses. Furthermore, due to the limitation of the NHANES database, we could not rule out biliary cirrhosis and primary liver diseases such as Wilson's disease and the use of steatogenic medication. Also, we could not evaluate the type of marijuana and the dose-response correlation between marijuana use vs. the prevalence of liver steatosis and ﬁbrosis. |
| Interpretation | 20 | Give a cautious overall interpretation of results considering objectives, limitations, multiplicity of analyses, results from similar studies, and other relevant evidence | 8,9 | The endocannabinoid system (ECS) has been established as a critical homeostatic regulator that acts through CB-1 and CB-2 receptors, promoting anti-fibrotic and anti-liver fat formation effects, respectively(6, 28). Marijuana contains >100 active cannabinoids(29) that interact with the same receptors for ECS to produce similar biological effects(30). The product of marijuana tetrahydrocannabinol decreases the CB1 density and lowers the overall CB1 activity. The other components of marijuana, such as cannabidiol and tetrahydrocannabivarin, reduce the activation of CB1, thereby decreasing liver steatosis and fibrosis. Since ﬁbrosis has a low prevalence in our population, marijuana use might not tip the balance of ECS. |
| Generalisability | 21 | Discuss the generalisability (external validity) of the study results |  |  |
| Other information | |  | | |
| Funding | 22 | Give the source of funding and the role of the funders for the present study and, if applicable, for the original study on which the present article is based | 9 | This study did not receive any specific grant from funding agencies in the public, commercial, or not-for-profit sectors. |

*Give information separately for cases and controls in case-control studies and, if applicable, for exposed and unexposed groups in cohort and cross-sectional studies.

**Note:** An Explanation and Elaboration article discusses each checklist item and gives methodological background and published examples of transparent reporting. The STROBE checklist is best used in conjunction with this article (freely available on the Web sites of PLoS Medicine at http://www.plosmedicine.org/, Annals of Internal Medicine at http://www.annals.org/, and Epidemiology at http://www.epidem.com/). Information on the STROBE Initiative is available at www.strobe-statement.org.
