# Supplementary material for: Marijuana use is inversely associated with liver steatosis detected by transient elastography in the general United States population in NHANES 2017–2018: A cross-sectional study
Source: PLoS One. 2023 May 18;18(5):e0284859. doi: 10.1371/journal.pone.0284859 (PMC10194898; doi:10.1371/journal.pone.0284859)
Supplement: Attachment — Submitted filename: Response to Reviewers.docx [file pone.0284859.s003.docx]

Dear Saral Rajiv Desai, Dear reviewers

Thank you for your letter and the reviewers' comments concerning our manuscript "Marijuana Use is Inverse Associated with Liver Steatosis Detected by Transient Elastography in the General United States Population in NHANES 2017-2018: A Cross-sectional Study" (PONE-D-23-02918). Those comments are all valuable and helpful for revising and improving our paper, as well as the important guiding significance to our research. We have studied the comments carefully and made corrections, which we hope to meet with approval. Revised portions are marked in red in the paper.

In addition to our point-by-point replies to the reviewers' comments, we also did our best to meet the standards of required editorial corrections and have made all changes easily identifiable. We hope that our revised manuscript meets your requirements. We upload our study’s minimal data set as a Supporting Information file. If any further action is needed, please let us know immediately.

We look forward to hearing back from you.

Sincerely, Authors,

**Reviewer #1:**

*1. Consider changing the manuscript title as follows: Marijuana use is inverseLY associated..."*

Response 1: Thank you for your suggestion. We changed the title to "Marijuana Use is Inversely Associated with Liver Steatosis Detected by Transient Elastography in the General United States Population in NHANES 2017-2018: A Cross-sectional Study".

*2. NHANES data are from a complex survey. As per CDC guidance, appropriate sampling weights should be used in all statistical analyses.*

Response 2: We sincerely appreciate for your advice. We totally agree with the importance of sound analytical approach and consideration of weighting in survey analysis. We have tried many times to re-analyze our surveys, but the results have been inconsistent. We are not sure if this has anything to do with the effect of cutoff values or weights amplifying a small number of missing covariates. So in this study, we just intended to use individual data to assess a cross-sectional study of marijuana use behaviors.

*3. I suggest adjusting the models for BMI. Please amend*

Response 3: We are grateful for the suggestion. Combining your and reviewer 2' suggestions, we adjusted the models for BMI and CKD in our analyses (Table 4). The association was unchanged after additionally adjusting for BMI and CKD.

**Table 4** Multivariate-adjusted logistic regression associations of marijuana use and steatosis/fibrosis

|  | n | Crude | | Model I | | Model II | | Model III | |
| --- | --- | --- | --- | --- | --- | --- | --- | --- | --- |
|  |  | **OR (95% CI) *P*** | | **OR (95% CI) *P*** | | **OR (95% CI) *P*** | | **OR (95% CI)** *P* | |
| **Steatosis** |  |  |  |  |  |  |  |  |  |
| Never | 1204 | 1(Ref) |  | 1(Ref) |  | 1(Ref) |  | 1(Ref) |  |
| Past user | 917 | 0.85 (0.72~1.01) | 0.072 | 0.87 (0.73~1.04) | 0.131 | 0.81 (0.63~1.06) | 0.127 | 0.83 (0.63~1.09) | 0.184 |
| Current user | 501 | 0.56 (0.45~0.70) | <0.001 | 0.62 (0.49~0.78) | <0.001 | 0.7 (0.5~0.98) | 0.039 | 0.69 (0.48~0.997) | 0.048 |
| **Fibrosis** |  |  |  |  |  |  |  |  |  |
| Never | 1204 | 1(Ref) |  | 1(Ref) |  | 1(Ref) |  | 1(Ref) |  |
| Past user | 917 | 0.91 (0.66~1.25) | 0.555 | 0.94 (0.68~1.3) | 0.712 | 0.86 (0.56~1.33) | 0.503 | 0.9 (0.57~1.41) | 0.64 |
| Current user | 501 | 0.69 (0.45~1.05) | 0.084 | 0.81 (0.52~1.25) | 0.338 | 0.82 (0.46~1.47) | 0.509 | 0.69 (0.36~1.3) | 0.248 |

*Model I: Adjusted for age and sex.*

*Model II: Adjusted for Model I +BMI +ethnicity + education level + marital status + poverty level+ Smoking status + alcohol intake.*

*Model III: Adjusted for Model II + CVD +CKD +COPD + hypertension + diabetes + anemia+ hepatitis B + hepatitis C.*

*Notes: OR, odds ratio; 95% CI, 95% confidence interval.*

*Abbreviations: BMI, body mass index; CVD, Cardiovascular disease; CKD, Chronic kidney disease; COPD, chronic obstructive pulmonary disease.*

**Reviewer #2:**

*1. It is my understanding that the analysis was performed without considering parameters of adiposity (BMI or waist circumference). It is well known that they are tightly related to fatty liver and to higher risk of fibrosis and in turn it is reported by the authors that the use of marijuana is associated with overweight/obesity. My advice it to test whether the independent association with use of cannabinoid remains significant adjusting for parameters of body adiposity*

Response 1: Thank you for your comments. We have adjusted the models for BMI (normal weight, overweight, and obese) in our analyses (Table 4). The association was unchanged after additionally adjusting for the BMI category.

*2. Similarly, I am wondering whether data about indirect biomarkers of insulin resistance (HOMA-IR) were considered as pathogenic modulator of the association*

Response 2: We sincerely appreciate for your advice. We added the HOMA-IR for analysis. Univariate analysis showed that HOMA-IR was associated with steatosis (OR: 1.44, 95% CI: 1.36–1.53, *P*<0.001) and fibrosis (OR: 1.1, 95% CI: 1.06–1.13, *P*<0.001). After adjusting for HOMA-IR in multivariate logistic regression models, only the association between current marijuana use with steatosis changed (OR: 0.61, 95% CI: 0.35–1.05, *P=*0.074, **Table S1**). Perhaps it is related to the setting of our model, and further exploration of this variable can be considered in subsequent models more related to insulin resistance.

**Table S1** Multivariate-adjusted logistic regression associations of marijuana use and steatosis/fibrosis

|  | n | Crude | | Model I | | Model II | | Model III | |
| --- | --- | --- | --- | --- | --- | --- | --- | --- | --- |
|  |  | **OR (95% CI) *P*** | | **OR (95% CI) *P*** | | **OR (95% CI) *P*** | | **OR (95% CI)** *P* | |
| **Steatosis** |  |  |  |  |  |  |  |  |  |
| Never | 1204 | 1(Ref) |  | 1(Ref) |  | 1(Ref) |  | 1(Ref) |  |
| Past user | 917 | 0.85 (0.72~1.01) | 0.072 | 0.87 (0.73~1.04) | 0.131 | 0.81 (0.63~1.06) | 0.127 | 0.74 (0.49~1.12) | 0.155 |
| Current user | 501 | 0.56 (0.45~0.70) | <0.001 | 0.62 (0.49~0.78) | <0.001 | 0.7 (0.5~0.98) | 0.039 | 0.61 (0.35~1.05) | 0.074 |
| **Fibrosis** |  |  |  |  |  |  |  |  |  |
| Never | 1204 | 1(Ref) |  | 1(Ref) |  | 1(Ref) |  | 1(Ref) |  |
| Past user | 917 | 0.91 (0.66~1.25) | 0.555 | 0.94 (0.68~1.3) | 0.712 | 0.86 (0.56~1.33) | 0.503 | 0.52 (0.24~1.1) | 0.085 |
| Current user | 501 | 0.69 (0.45~1.05) | 0.084 | 0.81 (0.52~1.25) | 0.338 | 0.82 (0.46~1.47) | 0.509 | 0.73 (0.28~1.88) | 0.508 |

*Model I: Adjusted for age and sex.*

*Model II: Adjusted for Model I +BMI +ethnicity + education level + marital status + poverty level+ Smoking status + alcohol intake.*

*Model III: Adjusted for Model II + CVD +CKD +COPD + hypertension + diabetes + anemia+ hepatitis B + hepatitis C + HOMA-IR*

*Abbreviations: BMI, body mass index; CVD, Cardiovascular disease; CKD, Chronic kidney disease; COPD, chronic obstructive pulmonary disease; CRP, C-Reactive Protein.*

*3. Following the same line of pathogenic reasoning I am wondering whether biomerkers of low grade inflammation are available*

Response 3: Thank you very much for your comments and professional advice. We added the C-reactive protein (CRP), which is representative of low grade inflammation, for further analysis. Univariate analysis showed that CRP was associated with steatosis (OR: 1.06, 95% CI: 1.04–1.08, *P*<0.001) and fibrosis (OR: 1.03, 95% CI: 1.02–1.05, *P*<0.001). After adjusting for CRP in multivariate logistic regression models, the association between current marijuana use with steatosis changed (OR: 0.71, 95% CI: 0.49–1.02, *P=*0.065, **Table S2**). If it is necessary to further clarify the correlation, perhaps this can be mentioned as a direction for future research**.**

**Table S2** Multivariate-adjusted logistic regression associations of marijuana use and steatosis/fibrosis

|  | n | Crude | | Model I | | Model II | | Model III | |
| --- | --- | --- | --- | --- | --- | --- | --- | --- | --- |
|  |  | **OR (95% CI) *P*** | | **OR (95% CI) *P*** | | **OR (95% CI) *P*** | | **OR (95% CI)** *P* | |
| **Steatosis** |  |  |  |  |  |  |  |  |  |
| Never | 1204 | 1(Ref) |  | 1(Ref) |  | 1(Ref) |  | 1(Ref) |  |
| Past user | 917 | 0.85 (0.72~1.01) | 0.072 | 0.87 (0.73~1.04) | 0.131 | 0.81 (0.63~1.06) | 0.127 | 0.83 (0.63~1.1) | 0.19 |
| Current user | 501 | 0.56 (0.45~0.70) | <0.001 | 0.62 (0.49~0.78) | <0.001 | 0.7 (0.5~0.98) | 0.039 | 0.71 (0.49~1.02) | 0.065 |
| **Fibrosis** |  |  |  |  |  |  |  |  |  |
| Never | 1204 | 1(Ref) |  | 1(Ref) |  | 1(Ref) |  | 1(Ref) |  |
| Past user | 917 | 0.91 (0.66~1.25) | 0.555 | 0.94 (0.68~1.3) | 0.712 | 0.86 (0.56~1.33) | 0.503 | 0.93 (0.59~1.47) | 0.751 |
| Current user | 501 | 0.69 (0.45~1.05) | 0.084 | 0.81 (0.52~1.25) | 0.338 | 0.82 (0.46~1.47) | 0.509 | 0.7 (0.37~1.34) | 0.286 |

*Model I: Adjusted for age and sex.*

*Model II: Adjusted for Model I + BMI +ethnicity + education level + marital status + poverty level+ Smoking status + alcohol intake.*

*Model III: Adjusted for Model II + CVD + CKD +COPD + hypertension + diabetes + anemia+ hepatitis B + hepatitis C + CRP*

*Abbreviations: BMI, body mass index; CVD, Cardiovascular disease; CKD, Chronic kidney disease; COPD, chronic obstructive pulmonary disease; CRP, C-Reactive Protein.*

*4. In addition, chronic kidney disease may also be associated with fatty liver and fibrosis, especially the albuminuric phenotype. These adjustments should be performed as well*

Response 4: We are grateful for the suggestion. After adjusting for CKD, the association was unchanged (Table 4).

We added urine albumin as a continuous variable for analysis. Univariate analysis revealed that urine albumin was associated with steatosis (OR: 0.57, 95% CI: 0.44–0.72, *P*<0.001) and fibrosis (OR: 0.39, 95% CI: 0.25–0.61, *P*<0.001) in participants. After adjusting for urine albumin in multivariate logistic regression models, the association was unchanged (**Table S3**).

**Table S3** Multivariate-adjusted logistic regression associations of marijuana use and steatosis/fibrosis

|  | n | Crude | | Model I | | Model II | | Model III | |
| --- | --- | --- | --- | --- | --- | --- | --- | --- | --- |
|  |  | **OR (95% CI) *P*** | | **OR (95% CI) *P*** | | **OR (95% CI) *P*** | | **OR (95% CI)** *P* | |
| **Steatosis** |  |  |  |  |  |  |  |  |  |
| Never | 1204 | 1(Ref) |  | 1(Ref) |  | 1(Ref) |  | 1(Ref) |  |
| Past user | 917 | 0.85 (0.72~1.01) | 0.072 | 0.87 (0.73~1.04) | 0.131 | 0.81 (0.63~1.06) | 0.127 | 0.83 (0.63~1.09) | 0.178 |
| Current user | 501 | 0.56 (0.45~0.70) | <0.001 | 0.62 (0.49~0.78) | <0.001 | 0.7 (0.5~0.98) | 0.039 | 0.69 (0.48~0.998) | 0.048 |
| **Fibrosis** |  |  |  |  |  |  |  |  |  |
| Never | 1204 | 1(Ref) |  | 1(Ref) |  | 1(Ref) |  | 1(Ref) |  |
| Past user | 917 | 0.91 (0.66~1.25) | 0.555 | 0.94 (0.68~1.3) | 0.712 | 0.86 (0.56~1.33) | 0.503 | 0.9 (0.57~1.42) | 0.654 |
| Current user | 501 | 0.69 (0.45~1.05) | 0.084 | 0.81 (0.52~1.25) | 0.338 | 0.82 (0.46~1.47) | 0.509 | 0.68 (0.36~1.3) | 0.246 |

*Model I: Adjusted for age and sex.*

*Model II: Adjusted for Model I + BMI +ethnicity + education level + marital status + poverty level+ Smoking status + alcohol intake.*

*Model III: Adjusted for Model II + CVD + CKD +COPD + hypertension + diabetes + anemia+ hepatitis B + hepatitis C + Urine albumin*

*Abbreviations: BMI, body mass index; CVD, Cardiovascular disease; CKD, Chronic kidney disease; COPD, chronic obstructive pulmonary disease.*
