# Supplementary material for: Marijuana use is inversely associated with liver steatosis detected by transient elastography in the general United States population in NHANES 2017–2018: A cross-sectional study
Source: PLoS One. 2023 May 18;18(5):e0284859. doi: 10.1371/journal.pone.0284859 (PMC10194898; doi:10.1371/journal.pone.0284859)
Supplement: Peer review history

# Peer review: Decision Letter 0

**DOI:** 10.1371/journal.pone.0284859.r001

**Authors:** Saral Rajiv Desai

12 Mar 2023

PONE-D-23-02918Marijuana Use is Inverse Associated with Liver Steatosis Detected by Transient Elastography in the General United States Population in NHANES 2017-2018: A Cross-sectional StudyPLOS ONE

Dear Dr. Yang,

Thank you for submitting your manuscript to PLOS ONE. After careful consideration, we feel that it has merit but does not fully meet PLOS ONE’s publication criteria as it currently stands. Therefore, we invite you to submit a revised version of the manuscript that addresses the points raised during the review process.

Please submit your revised manuscript by Apr 26 2023 11:59PM. If you will need more time than this to complete your revisions, please reply to this message or contact the journal office at plosone@plos.org. When you're ready to submit your revision, log on to https://www.editorialmanager.com/pone/ and select the 'Submissions Needing Revision' folder to locate your manuscript file.

Please include the following items when submitting your revised manuscript:

- A rebuttal letter that responds to each point raised by the academic editor and reviewer(s). You should upload this letter as a separate file labeled 'Response to Reviewers'.
- A marked-up copy of your manuscript that highlights changes made to the original version. You should upload this as a separate file labeled 'Revised Manuscript with Track Changes'.
- An unmarked version of your revised paper without tracked changes. You should upload this as a separate file labeled 'Manuscript'.

If you would like to make changes to your financial disclosure, please include your updated statement in your cover letter. Guidelines for resubmitting your figure files are available below the reviewer comments at the end of this letter.

If applicable, we recommend that you deposit your laboratory protocols in protocols.io to enhance the reproducibility of your results. Protocols.io assigns your protocol its own identifier (DOI) so that it can be cited independently in the future. For instructions see: https://journals.plos.org/plosone/s/submission-guidelines#loc-laboratory-protocols. Additionally, PLOS ONE offers an option for publishing peer-reviewed Lab Protocol articles, which describe protocols hosted on protocols.io. Read more information on sharing protocols at https://plos.org/protocols?utm_medium=editorial-email&utm_source=authorletters&utm_campaign=protocols.

We look forward to receiving your revised manuscript.

Kind regards,

Saral Rajiv Desai, M.D., C.C.R.P, C.C.R.C

Academic Editor

PLOS ONE

Journal Requirements:

When submitting your revision, we need you to address these additional requirements.

1. Please ensure that your manuscript meets PLOS ONE's style requirements, including those for file naming. The PLOS ONE style templates can be found at 

https://journals.plos.org/plosone/s/file?id=wjVg/PLOSOne_formatting_sample_main_body.pdf and 

https://journals.plos.org/plosone/s/file?id=ba62/PLOSOne_formatting_sample_title_authors_affiliations.pdf

2. We suggest you thoroughly copyedit your manuscript for language usage, spelling, and grammar. If you do not know anyone who can help you do this, you may wish to consider employing a professional scientific editing service. 

Whilst you may use any professional scientific editing service of your choice, PLOS has partnered with both American Journal Experts (AJE) and Editage to provide discounted services to PLOS authors. Both organizations have experience helping authors meet PLOS guidelines and can provide language editing, translation, manuscript formatting, and figure formatting to ensure your manuscript meets our submission guidelines. To take advantage of our partnership with AJE, visit the AJE website (http://learn.aje.com/plos/) for a 15% discount off AJE services. To take advantage of our partnership with Editage, visit the Editage website (www.editage.com) and enter referral code PLOSEDIT for a 15% discount off Editage services.  If the PLOS editorial team finds any language issues in text that either AJE or Editage has edited, the service provider will re-edit the text for free.

Upon resubmission, please provide the following:

The name of the colleague or the details of the professional service that edited your manuscript

A copy of your manuscript showing your changes by either highlighting them or using track changes (uploaded as a *supporting information* file)

A clean copy of the edited manuscript (uploaded as the new *manuscript* file)

3. In your Data Availability statement, you have not specified where the minimal data set underlying the results described in your manuscript can be found. PLOS defines a study's minimal data set as the underlying data used to reach the conclusions drawn in the manuscript and any additional data required to replicate the reported study findings in their entirety. All PLOS journals require that the minimal data set be made fully available. For more information about our data policy, please see http://journals.plos.org/plosone/s/data-availability.

Upon re-submitting your revised manuscript, please upload your study’s minimal underlying data set as either Supporting Information files or to a stable, public repository and include the relevant URLs, DOIs, or accession numbers within your revised cover letter. For a list of acceptable repositories, please see http://journals.plos.org/plosone/s/data-availability#loc-recommended-repositories. Any potentially identifying patient information must be fully anonymized.

Important: If there are ethical or legal restrictions to sharing your data publicly, please explain these restrictions in detail. Please see our guidelines for more information on what we consider unacceptable restrictions to publicly sharing data: http://journals.plos.org/plosone/s/data-availability#loc-unacceptable-data-access-restrictions. Note that it is not acceptable for the authors to be the sole named individuals responsible for ensuring data access.

We will update your Data Availability statement to reflect the information you provide in your cover letter.

4. We note that you have stated that you will provide repository information for your data at acceptance. Should your manuscript be accepted for publication, we will hold it until you provide the relevant accession numbers or DOIs necessary to access your data. If you wish to make changes to your Data Availability statement, please describe these changes in your cover letter and we will update your Data Availability statement to reflect the information you provide.

5. PLOS requires an ORCID iD for the corresponding author in Editorial Manager on papers submitted after December 6th, 2016. Please ensure that you have an ORCID iD and that it is validated in Editorial Manager. To do this, go to ‘Update my Information’ (in the upper left-hand corner of the main menu), and click on the Fetch/Validate link next to the ORCID field. This will take you to the ORCID site and allow you to create a new iD or authenticate a pre-existing iD in Editorial Manager. Please see the following video for instructions on linking an ORCID iD to your Editorial Manager account: https://www.youtube.com/watch?v=_xcclfuvtxQ

6. Please include your full ethics statement in the ‘Methods’ section of your manuscript file. In your statement, please include the full name of the IRB or ethics committee who approved or waived your study, as well as whether or not you obtained informed written or verbal consent. If consent was waived for your study, please include this information in your statement as well. 

Reviewers' comments:

Reviewer's Responses to Questions

**Comments to the Author**

1. Is the manuscript technically sound, and do the data support the conclusions?

The manuscript must describe a technically sound piece of scientific research with data that supports the conclusions. Experiments must have been conducted rigorously, with appropriate controls, replication, and sample sizes. The conclusions must be drawn appropriately based on the data presented. 

Reviewer #1: Partly

Reviewer #2: Yes

**********

2. Has the statistical analysis been performed appropriately and rigorously? 

Reviewer #1: Yes

Reviewer #2: I Don't Know

**********

3. Have the authors made all data underlying the findings in their manuscript fully available?

The PLOS Data policy requires authors to make all data underlying the findings described in their manuscript fully available without restriction, with rare exception (please refer to the Data Availability Statement in the manuscript PDF file). The data should be provided as part of the manuscript or its supporting information, or deposited to a public repository. For example, in addition to summary statistics, the data points behind means, medians and variance measures should be available. If there are restrictions on publicly sharing data—e.g. participant privacy or use of data from a third party—those must be specified.

Reviewer #1: Yes

Reviewer #2: Yes

**********

4. Is the manuscript presented in an intelligible fashion and written in standard English?

PLOS ONE does not copyedit accepted manuscripts, so the language in submitted articles must be clear, correct, and unambiguous. Any typographical or grammatical errors should be corrected at revision, so please note any specific errors here.

Reviewer #1: Yes

Reviewer #2: Yes

**********

5. Review Comments to the Author

Please use the space provided to explain your answers to the questions above. You may also include additional comments for the author, including concerns about dual publication, research ethics, or publication ethics. (Please upload your review as an attachment if it exceeds 20,000 characters)​

Reviewer #1: In the present manuscript the Authors report the results of a cross-sectional study investigating the association between marijuana use and liver steatosis and fibrosis detected through VCTE in the general US population using data from the recent 2017-2018 NHANES database.

The topic is certainly of interest and not frequently studied. I have the following comments:

1. Consider changing the manuscript title as follows: Marijuana use is inverseLY associated..."

2. NHANES data are from a complex survey. As per CDC guidance, appropriate sampling weights should be used in all statistical analyses.

3. I suggest adjusting the models for BMI. Please amend

Reviewer #2: The paper by Du R et al is cross-sectional analysis of NHANES round in which transient elastography was performed in the general population and indirect evidence of fatty liver content (CAP) and fibrosis (LSM) could be achieved and associated with self-reporting of marijuana use.

In spite of a lack of univariate association, when the analysis was performed adjusting for many relevant variables, the use of marijuana was associate with lower risk of liver steatosis but no association could be detected with liver fibrosis

Specific comments

1) It is my understanding that the analysis was performed without considering parameters of adiposity (BMI or waist circumference). It is well known that they are tightly related to fatty liver and to higher risk of fibrosis and in turn it is reported by the authors that the use of marijuana is associated with overweight/obesity. My advice it to test whether the independent association with use of cannabinoid remains significant adjusting for parameters of body adiposity

2) Similarly, I am wondering whether data about indirect biomarkers of insulin resistance (HOMA-IR) were considered as pathogenic modulator of the association

3) Following the same line of pathogenic reasoning I am wondering whether biomerkers of low grade inflammation are available

4) In addition, chronic kidney disease may also be associated with fatty liver and fibrosis, especially the albuminuric phenotype. These adjustments should be performed as well

**********

6. PLOS authors have the option to publish the peer review history of their article (what does this mean?). If published, this will include your full peer review and any attached files.

If you choose “no”, your identity will remain anonymous but your review may still be made public.

**Do you want your identity to be public for this peer review?** For information about this choice, including consent withdrawal, please see our Privacy Policy.

Reviewer #1: No

Reviewer #2: No

**********

[NOTE: If reviewer comments were submitted as an attachment file, they will be attached to this email and accessible via the submission site. Please log into your account, locate the manuscript record, and check for the action link "View Attachments". If this link does not appear, there are no attachment files.]

While revising your submission, please upload your figure files to the Preflight Analysis and Conversion Engine (PACE) digital diagnostic tool, https://pacev2.apexcovantage.com/. PACE helps ensure that figures meet PLOS requirements. To use PACE, you must first register as a user. Registration is free. Then, login and navigate to the UPLOAD tab, where you will find detailed instructions on how to use the tool. If you encounter any issues or have any questions when using PACE, please email PLOS at figures@plos.org. Please note that Supporting Information files do not need this step.

# Author comment: Author response to Decision Letter 0

**DOI:** 10.1371/journal.pone.0284859.r002

22 Mar 2023

Reviewer #1: 

1. Consider changing the manuscript title as follows: Marijuana use is inverseLY associated..."

Response 1: Thank you for your suggestion. We changed the title to "Marijuana Use is Inversely Associated with Liver Steatosis Detected by Transient Elastography in the General United States Population in NHANES 2017-2018: A Cross-sectional Study".

2. NHANES data are from a complex survey. As per CDC guidance, appropriate sampling weights should be used in all statistical analyses.

Response 2: We sincerely appreciate for your advice. We totally agree with the importance of sound analytical approach and consideration of weighting in survey analysis. We have tried many times to re-analyze our surveys, but the results have been inconsistent. We are not sure if this has anything to do with the effect of cutoff values or weights amplifying a small number of missing covariates. So in this study, we just intended to use individual data to assess a cross-sectional study of marijuana use behaviors.

3. I suggest adjusting the models for BMI. Please amend

Response 3: We are grateful for the suggestion. Combining your and reviewer 2' suggestions, we adjusted the models for BMI and CKD in our analyses (Table 4). The association was unchanged after additionally adjusting for BMI and CKD.

Reviewer #2:

1. It is my understanding that the analysis was performed without considering parameters of adiposity (BMI or waist circumference). It is well known that they are tightly related to fatty liver and to higher risk of fibrosis and in turn it is reported by the authors that the use of marijuana is associated with overweight/obesity. My advice it to test whether the independent association with use of cannabinoid remains significant adjusting for parameters of body adiposity

Response 1: Thank you for your comments. We have adjusted the models for BMI (normal weight, overweight, and obese) in our analyses (Table 4). The association was unchanged after additionally adjusting for the BMI category.

2. Similarly, I am wondering whether data about indirect biomarkers of insulin resistance (HOMA-IR) were considered as pathogenic modulator of the association

Response 2: We sincerely appreciate for your advice. We added the HOMA-IR for analysis. Univariate analysis showed that HOMA-IR was associated with steatosis (OR: 1.44, 95% CI: 1.36–1.53, P<0.001) and fibrosis (OR: 1.1, 95% CI: 1.06–1.13, P<0.001). After adjusting for HOMA-IR in multivariate logistic regression models, only the association between current marijuana use with steatosis changed (OR: 0.61, 95% CI: 0.35–1.05, P=0.074, Table S1). Perhaps it is related to the setting of our model, and further exploration of this variable can be considered in subsequent models more related to insulin resistance.

3. Following the same line of pathogenic reasoning I am wondering whether biomerkers of low grade inflammation are available

Response 3: Thank you very much for your comments and professional advice. We added the C-reactive protein (CRP), which is representative of low grade inflammation, for further analysis. Univariate analysis showed that CRP was associated with steatosis (OR: 1.06, 95% CI: 1.04–1.08, P<0.001) and fibrosis (OR: 1.03, 95% CI: 1.02–1.05, P<0.001). After adjusting for CRP in multivariate logistic regression models, the association between current marijuana use with steatosis changed (OR: 0.71, 95% CI: 0.49–1.02, P=0.065, Table S2). If it is necessary to further clarify the correlation, perhaps this can be mentioned as a direction for future research.

4. In addition, chronic kidney disease may also be associated with fatty liver and fibrosis, especially the albuminuric phenotype. These adjustments should be performed as well

Response 4: We are grateful for the suggestion. After adjusting for CKD, the association was unchanged (Table 4). 

We added urine albumin as a continuous variable for analysis. Univariate analysis revealed that urine albumin was associated with steatosis (OR: 0.57, 95% CI: 0.44–0.72, P<0.001) and fibrosis (OR: 0.39, 95% CI: 0.25–0.61, P<0.001) in participants. After adjusting for urine albumin in multivariate logistic regression models, the association was unchanged (Table S3).

Attachment Submitted filename: Response to Reviewers.docx Click here for additional data file.

# Peer review: Decision Letter 1

**DOI:** 10.1371/journal.pone.0284859.r003

**Authors:** Saral Rajiv Desai

10 Apr 2023

Marijuana Use is Inversely Associated with Liver Steatosis Detected by Transient Elastography in the General United States Population in NHANES 2017-2018: A Cross-sectional Study

PONE-D-23-02918R1

Dear Dr. Yang,

We’re pleased to inform you that your manuscript has been judged scientifically suitable for publication and will be formally accepted for publication once it meets all outstanding technical requirements.

Within one week, you’ll receive an e-mail detailing the required amendments. When these have been addressed, you’ll receive a formal acceptance letter and your manuscript will be scheduled for publication.

An invoice for payment will follow shortly after the formal acceptance. To ensure an efficient process, please log into Editorial Manager at http://www.editorialmanager.com/pone/, click the 'Update My Information' link at the top of the page, and double check that your user information is up-to-date. If you have any billing related questions, please contact our Author Billing department directly at authorbilling@plos.org.

If your institution or institutions have a press office, please notify them about your upcoming paper to help maximize its impact. If they’ll be preparing press materials, please inform our press team as soon as possible -- no later than 48 hours after receiving the formal acceptance. Your manuscript will remain under strict press embargo until 2 pm Eastern Time on the date of publication. For more information, please contact onepress@plos.org.

Kind regards,

Saral Rajiv Desai, M.D., C.C.R.P, C.C.R.C

Academic Editor

PLOS ONE

Additional Editor Comments (optional):

Reviewers' comments:

Reviewer's Responses to Questions

**Comments to the Author**

1. If the authors have adequately addressed your comments raised in a previous round of review and you feel that this manuscript is now acceptable for publication, you may indicate that here to bypass the “Comments to the Author” section, enter your conflict of interest statement in the “Confidential to Editor” section, and submit your "Accept" recommendation.

Reviewer #1: All comments have been addressed

**********

2. Is the manuscript technically sound, and do the data support the conclusions?

The manuscript must describe a technically sound piece of scientific research with data that supports the conclusions. Experiments must have been conducted rigorously, with appropriate controls, replication, and sample sizes. The conclusions must be drawn appropriately based on the data presented. 

Reviewer #1: Yes

**********

3. Has the statistical analysis been performed appropriately and rigorously? 

Reviewer #1: I Don't Know

**********

4. Have the authors made all data underlying the findings in their manuscript fully available?

The PLOS Data policy requires authors to make all data underlying the findings described in their manuscript fully available without restriction, with rare exception (please refer to the Data Availability Statement in the manuscript PDF file). The data should be provided as part of the manuscript or its supporting information, or deposited to a public repository. For example, in addition to summary statistics, the data points behind means, medians and variance measures should be available. If there are restrictions on publicly sharing data—e.g. participant privacy or use of data from a third party—those must be specified.

Reviewer #1: Yes

**********

5. Is the manuscript presented in an intelligible fashion and written in standard English?

PLOS ONE does not copyedit accepted manuscripts, so the language in submitted articles must be clear, correct, and unambiguous. Any typographical or grammatical errors should be corrected at revision, so please note any specific errors here.

Reviewer #1: Yes

**********

6. Review Comments to the Author

Please use the space provided to explain your answers to the questions above. You may also include additional comments for the author, including concerns about dual publication, research ethics, or publication ethics. (Please upload your review as an attachment if it exceeds 20,000 characters)

Reviewer #1: I congratulate the Authors for their thorough revision, which addressed all the points I previously raised. I have no further comments.

**********

7. PLOS authors have the option to publish the peer review history of their article (what does this mean?). If published, this will include your full peer review and any attached files.

If you choose “no”, your identity will remain anonymous but your review may still be made public.

**Do you want your identity to be public for this peer review?** For information about this choice, including consent withdrawal, please see our Privacy Policy.

Reviewer #1: No

**********

# Editor report: Acceptance letter

**DOI:** 10.1371/journal.pone.0284859.r004

**Authors:** Saral Rajiv Desai

10 May 2023

PONE-D-23-02918R1 

Marijuana Use is Inversely Associated with Liver Steatosis Detected by Transient Elastography in the General United States Population in NHANES 2017-2018: A Cross-sectional Study 

Dear Dr. Yang:

I'm pleased to inform you that your manuscript has been deemed suitable for publication in PLOS ONE. Congratulations! Your manuscript is now with our production department. 

If your institution or institutions have a press office, please let them know about your upcoming paper now to help maximize its impact. If they'll be preparing press materials, please inform our press team within the next 48 hours. Your manuscript will remain under strict press embargo until 2 pm Eastern Time on the date of publication. For more information please contact onepress@plos.org.

If we can help with anything else, please email us at plosone@plos.org. 

Thank you for submitting your work to PLOS ONE and supporting open access. 

Kind regards, 

PLOS ONE Editorial Office Staff

on behalf of

Dr. Saral Rajiv Desai 

Academic Editor

PLOS ONE
